# DNA hypermethylation associated with upregulated gene expression in prostate cancer demonstrates the diversity of epigenetic regulation

**DOI:** 10.1186/s12920-020-0657-6

**Published:** 2020-01-08

**Authors:** Ieva Rauluseviciute, Finn Drabløs, Morten Beck Rye

**Affiliations:** 1 Department of Clinical and Molecular Medicine, NTNU - Norwegian University of Science and Technology, P.O. Box 8905, NO-7491 Trondheim, Norway; 2 Clinic of Surgery, St. Olavs Hospital, Trondheim University Hospital, NO-7030 Trondheim, Norway

**Keywords:** Epigenetics, DNA methylation, Prostate cancer, Gene expression

## Abstract

**Background:**

Prostate cancer (PCa) has the highest incidence rates of cancers in men in western countries. Unlike several other types of cancer, PCa has few genetic drivers, which has led researchers to look for additional epigenetic and transcriptomic contributors to PCa development and progression. Especially datasets on DNA methylation, the most commonly studied epigenetic marker, have recently been measured and analysed in several PCa patient cohorts. DNA methylation is most commonly associated with downregulation of gene expression. However, positive associations of DNA methylation to gene expression have also been reported, suggesting a more diverse mechanism of epigenetic regulation. Such additional complexity could have important implications for understanding prostate cancer development but has not been studied at a genome-wide scale.

**Results:**

In this study, we have compared three sets of genome-wide single-site DNA methylation data from 870 PCa and normal tissue samples with multi-cohort gene expression data from 1117 samples, including 532 samples where DNA methylation and gene expression have been measured on the exact same samples. Genes were classified according to their corresponding methylation and expression profiles. A large group of hypermethylated genes was robustly associated with increased gene expression (UPUP group) in all three methylation datasets. These genes demonstrated distinct patterns of correlation between DNA methylation and gene expression compared to the genes showing the canonical negative association between methylation and expression (UPDOWN group). This indicates a more diversified role of DNA methylation in regulating gene expression than previously appreciated. Moreover, UPUP and UPDOWN genes were associated with different compartments — UPUP genes were related to the structures in nucleus, while UPDOWN genes were linked to extracellular features.

**Conclusion:**

We identified a robust association between hypermethylation and upregulation of gene expression when comparing samples from prostate cancer and normal tissue. These results challenge the classical view where DNA methylation is always associated with suppression of gene expression, which underlines the importance of considering corresponding expression data when assessing the downstream regulatory effect of DNA methylation.

## Background

Prostate cancer (PCa) is the second most common type of cancer in men worldwide and the most common cancer type in western countries [1–3]. PCa is a complex cancer, which displays large molecular heterogeneity in tumour foci from different patients, and also in different tumour foci from the same patient [4]. Unlike several other cancers, PCa demonstrates few distinct genetic drivers [5, 6], which has made it challenging to identify and study the mechanisms of PCa development and progression. However, studies of DNA methylation in PCa are providing new insights. DNA methylation is one of the key mechanisms of regulating gene transcription in cells, and changes in DNA methylation patterns can therefore play a crucial role in PCa [7]. DNA is methylated by transferring a methyl group form S-adenosyl-L-methionine to 5′ carbon atom of a cytosine in a CpG dinucleotide context, creating 5-methylcytosine (5mC) [8, 9]. CpGs tend to cluster into CpG islands (CGIs) — regions between 300 and 3000 bp in length with greater than 50% GC content and CpG/GpC ratio greater than 0.6 [8, 10, 11].

DNA methylation alterations are common in PCa, both as early events in cancer development and in more advanced tumours [7, 12]. For gene regulation, the generally accepted regulative relationship between DNA methylation and gene expression is that the promoters and intergenic regions of normally active tumour suppressor genes (TSGs) become hypermethylated in cancer, resulting in downregulated gene expression [9, 13, 14]. Accordingly, genomic regions associated with oncogenes become hypomethylated in order to activate their expression [9, 15]. Genome-wide hypomethylation is also often observed in cancer, particularly in repeat DNA sequences, but is less targeted to regulatory regions and CpG islands [15–17]. Overall, abnormal methylation of TSG promoters is a common hallmark for cancer (including PCa), which can help to describe cancer development and be utilized for cancer detection and prognosis [18–22].

Recent efforts combining genome-wide DNA methylation and gene expression analysis on the same samples enable studies of more subtle changes in DNA methylation and gene expression, beyond the classical dogma. Although gene silencing by promoter hypermethylation seems to be the most likely mode of action, there is growing evidence of a more complex view on the effect of DNA hypermethylation in various contexts [21, 23–25]. In particular, for genes that become hypermethylated, the associated expression level can be unaffected or even upregulated in some cases [25]. It has also been shown that certain genes with unmethylated CGIs in the promoter regions are unable to produce functional transcripts, because RNA Pol II is not recruited [26]. Local methylation of individual residues has shown to be significant for the regulation of expression, and is thus able to counteract the methylation status of the genomic region as a whole [24, 27]. Moreover, some transcription factors prefer to bind methylated rather than unmethylated CpGs [28–31]. Low density of 5mCs in the promoter region can prevent the binding of transcription machinery, even though the region as a whole is methylated, and the expression of sparsely (but not densely) methylated genes can be activated by enhancers [24, 32, 33]. The general mechanisms behind these patterns and observations are not well known.

**Fig. 1:**
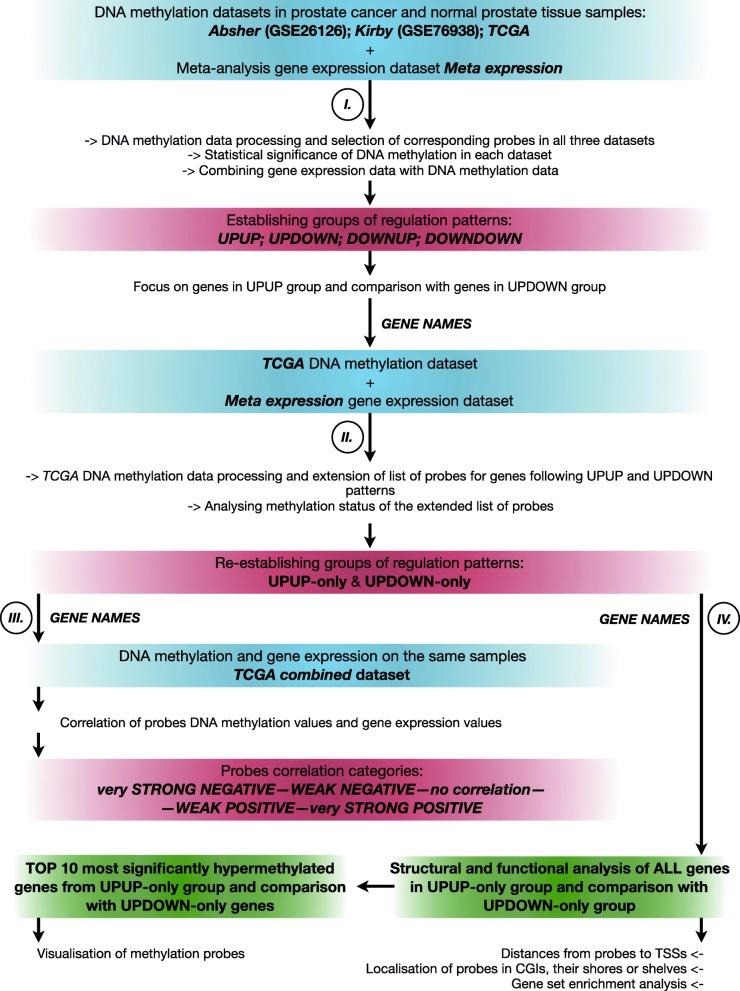
The complete workflow of the study. (I): Analysis initially used three DNA methylation datasets (Absher, Kirby and TCGA) and the Meta expression dataset of gene expression. Based on promoter methylation status and gene expression, four groups of gene regulation patterns were established, and genes were grouped accordingly. (II): The 450 K TCGA DNA methylation dataset was used to associate genes with more methylation probes to filter into UPUP-only and UPDOWN-only gene groups by removing genes with ambiguous DNA methylation statuses. (III): Correlation analysis was performed using TCGA combined dataset, where DNA methylation and gene expression values derived from the same samples were individually compared. (IV): UPUP-only and UPDOWN-only genes were studied genome-wide by analysing the distances between probes and TSSs of the associated genes, genomic locations of probes, and performing a GSEA. In addition, several individual genes were investigated by visualizing methylation probes

In this study we have performed an integrated analysis on three DNA methylation datasets with PCa and normal prostate tissue samples, one gene expression dataset aggregated over five cohorts comparing PCa and normal samples, and one dataset where DNA methylation and gene expression have been measured on the same tissue samples (Table 1, Fig. 1). We use these data to identify robust and reproducible associations between DNA methylation and gene expression. We demonstrate that DNA hypermethylation can be associated not only with downregulation of gene expression, but also that a considerable fraction of hypermethylated genes is associated with upregulation of gene expression. These two types of association share similarities in their methylation patterns and functional properties, but they also represent distinct groups with specific features, indicating a more diversified effect of DNA methylation on gene expression in PCa.

**Table 1:** DNA methylation and gene expression datasets used in the study

Dataset	Abbreviation in a text	Number of samples		Platform	Reference
		Cancer	Normal		
GSE26126 *(DNA methylation)*	*Absher*	95	86	Illumina Human Methylation 27 BeadChip array (27 k)	[22]
GSE76938 *(DNA methylation)*	*Kirby*	73	63	Illumina Infinium Human Methylation 450 BeadChip array (HM450)	[19]
The Cancer Genome Atlas (TCGA) *(DNA methylation)*	*TCGA*	503	50	Illumina Infinium Human Methylation 450 BeadChip array (HM450)	[34]
Meta-analysis gene expression dataset *(previously processed data)*	*Meta expression*	887	230	Various	[35]
TCGA *(gene expression data processed for this publication)*					[34]
TCGA *(DNA methylation together with gene expression)*	*TCGA combined*	497	35	Illumina Infinium Human Methylation 450 BeadChip array (HM450)	[34]

## Results

### DNA hypermethylation and upregulated gene expression is a robust association pattern

**Fig. 2:**
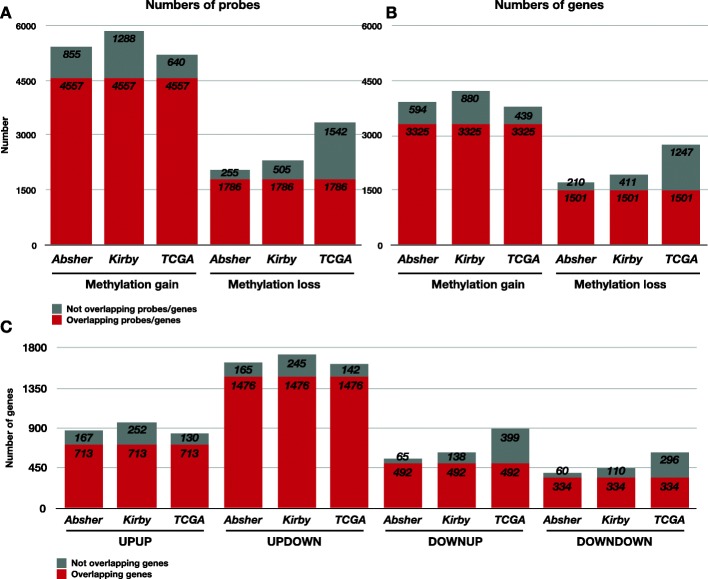
Number of genes and probes in the three DNA methylation datasets Absher, Kirby and TCGA with different DNA methylation and gene expression statuses in PCa compared to normal tissue samples. The resemblance between the datasets is high in terms of: **a** probes with gain and loss of methylation, **b** genes with gain and loss of methylation and **c** genes classified in the groups UPUP, UPDOWN, DOWNUP and DOWNDOWN based on correspondence between methylation and gene expression. Red indicates the fraction of overlapping probes/genes, while grey indicates non-overlapping probes/genes

To initially limit the number of methylation sites, we first analysed the dataset from *Absher* (Table 1), which focuses on promoter regions. Of the 27,578 DNA methylation probes analysed in *Absher*, we identified 6110 probes that gained methylation and 2916 that lost methylation when PCa samples were compared to normal samples. We then assessed the robustness of these differences by comparing them with corresponding PCa-to-normal changes in the datasets from *Kirby* and *TCGA* (Table 1). Among 11,375 corresponding probes with data in all three methylation datasets, 4557 were significantly hypermethylated in PCa compared to normal tissue samples, while 1786 were significantly hypomethylated (*p* < 0.05) in all three cohorts (Fig. 2a). These probes were associated with 3326 and 1502 genes, respectively (Fig. 2b). A few genes were recurrent among the top 5 most significantly hypermethylated. Genes *SOSTDC1* and *FLT4* are shared between the *Absher* and *Kirby* datasets, while the gene *CYBA* is shared between the *Absher* and *TCGA* datasets.

**Fig. 3:**
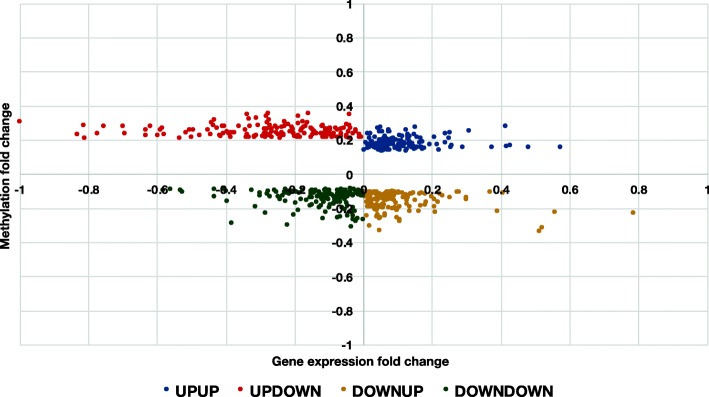
UPDOWN genes displayed higher methylation fold changes than UPUP genes. However, UPUP genes also demonstrate strong positive association between methylation and gene expression, supporting the additional relevance of the UPUP pattern in gene regulation. One hundred fifty genes from UPUP, UPDOWN, DOWNUP and DOWNDOWDN groups with the highest DNA methylation fold changes were selected, their gene expression and average DNA methylation fold changes visualized, where each data point represents one gene. Average methylation fold changes were calculated from all corresponding probes in Absher, Kirby and TCGA datasets

DNA methylation results were then combined with a dataset of previously identified robust gene expression changes in PCa [35] to distinguish four groups of regulation patterns (UPUP — methylation gain, expression upregulation; UPDOWN — methylation gain, expression downregulation; DOWNUP — methylation loss, expression upregulation; DOWNDOWN – methylation loss, expression downregulation). As expected, most genes (1476 overlapping genes in *Absher*, *Kirby* and *TCGA*, *p* < 0.05) followed the canonical pattern where hypermethylated promoters leads to downregulated expression (UPDOWN group, Fig. 2c). However, a large number of hypermethylated genes (713, *p* < 0.05) were associated with increased expression (UPUP group, Fig. 2c). These observations were similarly robust for UPUP and UPDOWN groups: on average 89% of the UPDOWN and 80% of the UPUP methylation changes were present in all three datasets (Additional file 1: Table S1). Genes from the UPDOWN group displayed on average higher methylation fold changes than genes form the UPUP group, and a higher negative impact on gene expression for a subset of genes (Fig. 3), supporting the UPDOWN pattern as the most important mode of regulation. However, UPUP genes also showed comparably strong positive association between DNA methylation and gene expression (Fig. 3), supporting the additional relevance of the UPUP pattern. Methylation changes are weaker and less abundant for the genes in DOWNUP and DOWNDOWN groups compared to the two groups with hypermethylation. Only 70% of methylation changes were present in all three datasets and with a noticeable poorer overlap in the TCGA dataset (Fig. 2, Additional file 1: Table S1). Average fold changes are also smaller for DOWNUP and DOWNDOWN genes (Fig. 3).

In the three DNA methylation datasets the majority of genes showed consistent association with either hypermethylated or hypomethylated probes (Additional file 1: Table S2). For genes associated with multiple probes (on average 266 and 697 genes in the UPUP and UPDOWN groups, respectively) less than 2% showed association with both hypermethylated and hypomethylated probes. Some of these genes, such as *GNAS* and *PEG10*, showed the same inconsistent associations in all three datasets. Inconsistency was higher in the DOWNUP and DOWNDOWN groups, with 2.21 and 6.81% of genes on average in three datasets with both hyper- and hypomethylated probes (Additional file 1: Table S2).

Since the UPUP genes was the non-canonical group with the most consistent non-canonical methylation/expression pattern, we decided to focus on the group of UPUP genes in the remaining part of this study. However, we also made a parallel analysis of UPDOWN genes to see how the UPUP group compared to the classical UPDOWN pattern in terms of robustness of the observed patterns.

### UPUP gene methylation patterns are robust when expanding the number of probe-gene associations with HM450 data

The *Absher* DNA methylation dataset has a significantly smaller number of probes, compared to the HM450 BeadChip used in *Kirby* and *TCGA*. To investigate further the methylation pattern around UPUP genes we extended our analysis to all gene-probe associations in the HM450 reference and compared UPUP and UPDOWN genes in this extended setting. This substantially increased the number of gene-probe associations (125,704 associations in total with an average of 16.4 probes per gene, compared to 11,375, with an average of 1.5 for the 27 k).

The initial set of UPUP genes was filtered according to the methylation patterns of their associated HM450 probes. All UPUP genes with at least one significantly (*p* < 0.05) downregulated methylation probe were removed, reducing the number of UPUP genes from 713 to 105. This UPUP-only group thus consists of genes which either has only upregulated methylation probes or a combination of upregulated and non-differentially expressed probes, but no downregulated methylation probes. The same strategy was applied to create an UPDOWN-only group of genes, reducing the number of UPDOWN genes from 1476 to 192. Genes in UPUP-only and UPDOWN-only groups have on average 9.5 and 9.8 hypermethylated probes per gene, respectively. Moreover, 78.10% of all UPUP-only genes have more than 50% of the associated probes hypermethylated, while the corresponding number for UPDOWN-only genes is 46.35%. In addition, 11.43% of UPUP-only genes have all associated probes consistently hypermethylated, compared to 7.29% of the UPDOWN-only genes. Thus, when increasing the number of methylation probes using HM450 data, we still observe comparable robustness of gene-probe associations in the UPUP-only and UPDOWN-only groups of genes. This strengthen the indication that the observed UPUP pattern constitute a biological relevant epigenetic layer of gene regulation. The two refined groups of genes (UPUP-only and UPDOWN-only) with unambiguous methylation patterns — no probes with methylation loss associated — were analysed further.

### Probes associated with UPUP-only genes demonstrate a distinct correlation pattern between DNA methylation and gene expression compared to probes associated with UPDOWN-only genes

The TCGA cohort contains gene expression and DNA methylation measured on the exact same samples (in this text defined as the *TCGA combined* dataset). This means that expression and methylation profiles are directly comparable, with minimal confounding by varying tumour content and tissue composition. We used the *TCGA combined* dataset to compare the strength of gene-probe associations for the UPUP-only and UPDOWN-only gene groups (105 and 192 genes, respectively) by calculating the Pearson correlation between *TCGA combined* methylation and expression profiles across all samples. The probes were assigned to different correlation groups, based on the strength and the sign of their correlation values (very strong negative to very strong positive correlation) (Additional file 1: Table S3).

**Fig. 4:**
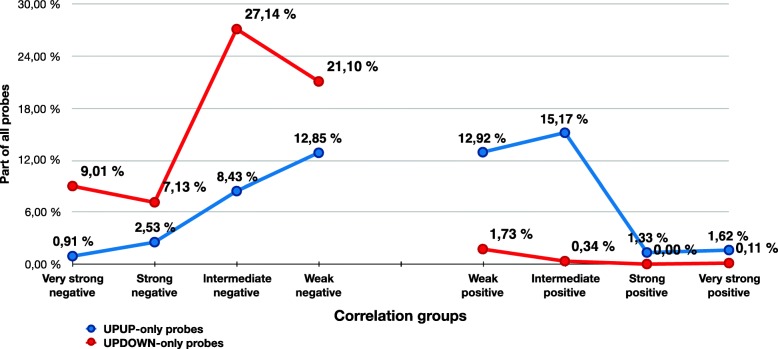
The UPUP-only group shows a weaker correspondence to the UPUP pattern compared to the UPDOWN-only group and the corresponding UPDOWN pattern, although a clear difference between two groups can be seen. Most of the UPDOWN-only DNA methylation probes are negatively correlated with the expression of corresponding genes, while a few are positively correlated positively. UPUP-only pattern includes some negatively correlated probes, but still the larger fraction shows positive correlation. Overall, the UPUP and UPDOWN patterns are clearly distinct

As expected, probes for the UPDOWN-only genes generally display a negative correlation, with most probes in the intermediate correlation group (27.14%) (Fig. 4, Additional file 1: Table S3), and only a small number of UPDOWN-only probes show a positive correlation. Correspondingly, most of the UPUP-only probes (15.17%) have intermediate positive correlation. However, genes in the UPUP-only group are also somewhat associated with weak and intermediate negatively correlated probes. Nevertheless, the differences observed in Fig. 4 demonstrate that the UPUP-only probes follow a distinct correlation pattern compared to UPDOWN-only probes, though the overall positive association between methylation and gene expression for UPUP-only probes is weaker than the corresponding anticorrelation for UPDOWN-only group of probes.

### UPUP-only probes are more closely associated with TSSs of the associated genes compared to UPDOWN-only probes

**Fig. 5:**
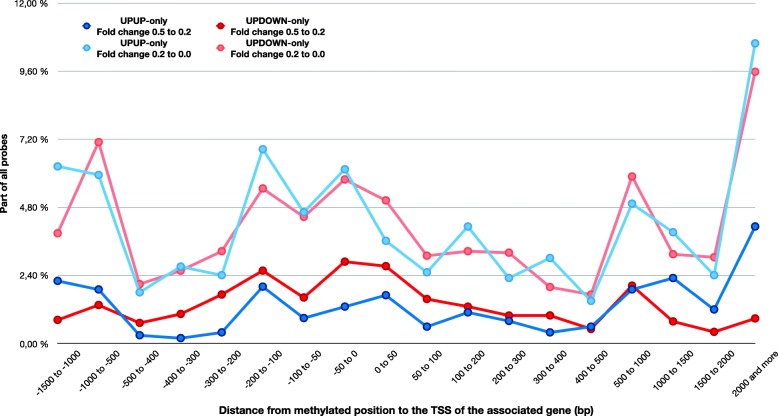
UPUP-only and UPDOWN-only genes show different distribution close to TSS for methylation probes with high fold changes. The distribution of probes with lower fold change is similar in the two groups (light blue for UPUP-only probes, light red for UPDOWN-only probes)

We calculated the distance between each hypermethylated probe and TSSs of the associated genes in the UPUP-only and UPDOWN-only groups, hypothesizing that sites closer to TSS might have a higher impact on the expression level than sites further away from TSS. When comparing calculated distances and average methylation fold change of the genes in each of the two groups, it is clear that there are more UPDOWN-only than UPUP-only probes with a higher fold change closer to the TSS, and that this is consistent across a region of at least +/− 400 bp around the TSS (Fig. 5, Additional file 1: Table S4). On the other hand, a far larger fraction of UPUP-only genes (57.14%) are enriched for hypermethylated probes most proximal to the TSS (+/− 50 bp), compared to UPDOWN-only genes (26.04%) (Additional file 1: Figure S1). The distribution of probes with a smaller fold change does not show any clear differences between the two groups (Fig. 5). More than 80% of all probes (both high and low fold changes) are located in the window of − 1500 to 1500 bp from the TSSs of the associated genes and all genes have at least one hypermethylated probe located in this region (Additional file 1: Figure S1). Somewhat fewer probes from both groups are located upstream from the TSS (46.01% of all UPUP-only probes and 47.42% of UPDOWN-only probes).

In addition, we checked the location of hypermethylated probes in the genome regions of particular importance to regulation of gene expression — CGIs, their shores or shelves. For UPUP-only group, 81.90% have significantly hypermethylated probes located in one of the three analysed genomic region types. This is higher than 73.44% for UPDOWN-only group. However, similar fractions of genes in both groups have hypermethylated probes in at least one of the three region types (Additional file 1: Table S5). Thus, if these three types of regions are indicative of regulatory potential, the observed similarities should indicate a comparable regulatory potential for both groups of genes. Taken together, the analysis of probe distances to TSS and genomic locations of the probes implies a robust regulatory relationship between DNA methylation and gene expression for both UPUP-only and UPDOWN-only groups of genes.

We also counted the fractions of methylation sites for genes in UPUP-only and UPDOWN-only groups found in 5’UTR, 3’UTR, exons, coding exons, introns, first exons, first coding exons and first introns (Table 2). The highest fractions of hypermethylated and non-differentially methylated UPUP-only and UPDOWN-only probes are located in exons and introns, but more than half of the probes are specifically located in the first exon and intron. UPDOWN-only genes have slightly more hypermethylated probes in first intron and exon than UPUP-only genes. However, the fraction of hypermethylated probes in the first coding exons are similar for UPDOWN-only and UPUP-only genes, suggesting that the main difference for UPDOWN-only hypermethylation is in 5’UTR exons. UPDOWN-genes also has a higher fraction of non-differentially methylated probes in the 3’UTR regions, supporting the slight bias of UPDOWN-only probes towards the beginning of the gene compared to UPUP-only probes. Apart from this, there are only subtle differences between UPDOWN-only and UPUP-only genes in their methylation site association to the gene region categories.

**Table 2:** Fractions of all hypermethylated and non-differentially methylated probes in the TCGA DNA methylation dataset for UPUP-only and UPDOWN-only regulation pattern groups, where probes are located in different genomic regions: 3’UTR, 5’UTR, coding exons, exons and introns of the associated genes. Furthermore, fractions of probes that are specifically located in the first coding exon, first exon and first intron is also shown

Region	UPUP-only		UPDOWN-only	
	Part of all hypermethylated probes located in a specific region	Part of all non-differentially methylated probes located in a specific	Part of all hypermethylated probes located in a specific region	Part of all non-differentially methylated probes located in a specific
3’UTR	9.66% (94 probes)	8.53% (36 probes)	7.02% (131 probes)	11.61% (88 probes)
5’UTR	14.25% (130 probes)	13.92% (55 probes	16.14% (297 probes)	13.72% (104 probes)
Coding exons	18.41% (181 probes)	17.26% (73 probes)	14.16% (266 probes)	14.19% (108 probes)
Exons	39.25% (389 probes)	36.49% (158 probes)	35.36% (664 probes)	35.09% (267 probes)
Introns	26.80% (194 probes)	24.57% (86 probes)	26.91% (465 probes)	32.47% (225 probes)
	Part of all hypermethylated probes in any coding exon/exon/intron	Part of all non-differentially methylated probes in any coding exon/exon/intron	Part of all hypermethylated probes in any coding exon/exon/intron	Part of all non-differentially methylated probes in any coding exon/exon/intron
First coding exon	64% (116 probes)	72.60% (53 probes)	63.53% (169 probes)	55.55% (60 probes)
First exon	66.84% (260 probes)	61.39% (97 probes)	70.03% (465 probes)	55.06% (147 probes)
First intron	70.62% (137 probes)	69.77% (60 probes)	79.14% (368 probes)	63.11% (142 probes)

### Genes in UPUP-only and UPDOWN-only groups are associated with the same regulatory mechanism, but affect genes in different cellular compartments

Genes from the UPUP-only and UPDOWN-only groups are associated with the same regulatory mechanism. Gene set enrichment analysis in Enrichr showed that both groups of genes were significantly associated with transcription factor SUZ12 in two categories; *‘ENCODE TF ChIP-seq 2015’* and *‘ENCODE and ChEA Consensus TFs from ChIP-X’* (*p* < 0.001), indicating possible involvement in this cellular regulatory network. Combined enrichment score from the *‘Consensus’* category for UPUP-only genes was lower compared to the score for UPDOWN-only (38.68 and 53.42, respectively). Results from *‘ENCODE Histone Modifications 2015’* enhance relations to regulatory functions, where both gene groups were linked to H3K27me3 histone modification, known to interact with (or is modulated by) the *Polycomb* complex, which also includes SUZ12.

Interestingly, the clearest difference between UPUP-only and UPDOWN-only genes was observed in the *‘Jensen COMPARTMENTS’* category. In this category the UPUP-only genes showed statistically significant association (*p* < 0.001) with terms related to nuclear chromatin, nucleosomes, DNA packaging and protein-DNA complexes. The combined enrichment scores of the top 5 most significant hits vary from 43.24 for *‘Nuclear_chromosome’* to 52.99 for *‘Nuclear_chromatin”*. In comparison, UPDOWN-only genes showed association with terms related to extracellular features, including extracellular exosome, vesicle, organelle, membrane-bounded vesicle and cytoskeletal component — type III intermediate filament. However, combined enrichment scores for top 5 hits were considerably higher for UPDOWN-only genes, ranging from 207.15 for *‘Extracellular_organelle’* to 325.07 for *‘Extracellular_region’*.

### Distribution of hypermethylated probes along UPUP-only genes is more complex compared to UPDOWN-only genes

We selected the top 10 most significantly hypermethylated UPUP-only and UPDOWN-only genes to investigate how the detailed distribution of methylation probes differ in the local genomic region surrounding these genes (Additional file 1: Table S6). Observing the top 10 genes from the canonical UPDOWN-only group, we spotted a clear trend; hypermethylated probes tend to form a cluster around the TSS of the associated genes, and this cluster usually overlaps with a CGI. The formation of clusters is here evaluated visually. The distances of probes to the TSS vary with majority being more distant than 50 bp. The distribution of the probes for UPDOWN-only genes can be distinguished according to how hypermethylated and non-differentially methylated probes distribute across the genes. Three genes (*PLA2G3*, *WFDC2* and *MFAP4*) have one or two additional significantly hypermethylated probes located away from the cluster, which are less significantly hypermethylated according to the *p*-value. Seven genes (*SCGB3A1*, *EFS*, *KLF8*, *COL3A1*, *TMEM106A*, *RGN* and *SPARCL1*) have one to three non-differentially methylated probes located outside the hypermethylated cluster.

**Fig. 6:**
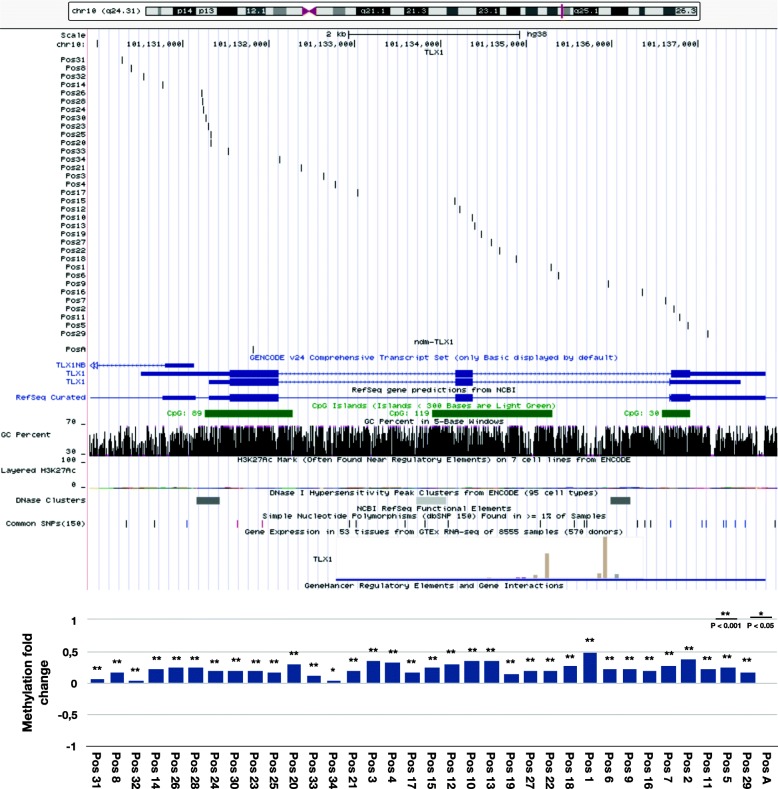
UCSC Genome Browser window for the gene TLX1 together with methylation fold changes for each visualized position in the same order. The distribution of probes associated with this gene is a distinctive example for the UPUP-only regulation pattern group. Thirty-four significantly hypermethylated probes cover three CGIs with a higher density for one of the TSSs. Pos1–34 stand for significantly (*p* < 0.001 and *p* < 0.05) hypermethylated probes from most (Pos1) to least (Pos34) significantly methylated probe. PosA is a non-differentially methylated probe

The ten most significant genes from the UPUP-only pattern are more challenging to group based on the distribution of the probes. However, two groups of genes with similar distribution of probes patterns can be distinguished. Five genes (*CPT1B*, *LTK*, *ZAR1*, *SRPX2* and *LRRC25*) are similar to the seven-gene UPDOWN-only pattern with a cluster of hypermethylated probes around TSS, and one to three non-differentially methylated probes located outside of the cluster. Three genes (*GSC*, *FEV* and *HIST1H3E*) do not display any clear clusters of hypermethylated probes and also have at least four non-differentially methylated probes associated, which show no systematic distribution pattern. However, hypermethylated probes for this group of genes do overlap with CGIs. The two last genes in UPUP-only group cannot be assigned to any clear pattern. The gene *TLX1* is associated with 34 significantly hypermethylated probes and one non-differentially methylated probe (Fig. 6). The gene has three CGIs, which are covered by three clusters of probes with a particularly dense cluster in one of the two TSSs of the gene. The other TSS is faintly covered with four hypermethylated probes, where two of them are among the least significantly hypermethylated (Pos31 and Pos32). In addition, DNA methylation fold changes are higher for a denser cluster that covers the second TSS. This distribution of methylation probes and the change in their methylation status could indicate a usage of an alternative TSS, which could explain the upregulated gene expression due to insufficient hypermethylation of an alternative TSS. However, DBTSS and ZENBU do not show any alternative TSSs for this gene in prostate or other tissues. The last gene, *TSPAN16*, is the only gene in UPUP-only top ten list that has five non-differentially methylated probes and only one very significantly hypermethylated, which also overlaps with the TSS. Overall, we observe that though distribution patterns for UPUP-only genes have similarities with the patterns for UPDOWN-only genes, the UPUP-only pattern is more difficult to generalize due to its higher complexity.

## Discussion

A great advantage of our study is the large amount of high-resolution and genome-wide data that has been integrated and analysed. We collected and analysed methylation data from 671 PCa and 199 normal prostate tissue samples in parallel with gene expression data from 887 cancer and 230 normal samples in order to associate methylation with gene expression and identified robust changes in both. Moreover, we also utilize in our study the analysis of the *TCGA combined* dataset with 532 PCa and normal tissue samples, where DNA methylation and gene expression have been measured on the exact same samples. This surpasses the amount of data that have been analysed previously in studies with similar scope, and the present study demonstrates changes in DNA methylation and gene expression that are robust when integrated across these large PCa cohorts.

Previous research on DNA methylation in PCa has concluded that promoter DNA hypermethylation of genes is the most frequent change when PCa is compared to normal tissue [19, 22, 36, 37]. We also observed promoter DNA hypermethylation more frequently than hypomethylation in all our comparisons (Fig. 2a-b). The mechanisms behind promoter DNA hypermethylation, how and why it appears in cancer, are still not well known. It has been proposed that promoter CGI-specific DNA hypermethylation can occur due to oxidative damage that forces the formation of specific silencing complexes, such as PRC4, found only in cancer and stem cells [38]. As we would expect, our Gene Set Enrichment Analysis (GSEA) of robustly hypermethylated genes revealed relationships with histone modification H3K27me3 and the protein SUZ12, which is a partner of EZH2 (repeatedly reported to play a role in PCa development and progression) in PRC2, and its overexpression is also known to be involved in carcinogenesis [39–43]. For both UPUP and UPDOWN genes such associations with Polycomb were observed.

Suppression of gene expression has been considered as the main function of promoter region DNA hypermethylation. Description of other possible regulatory effects are sparse and have mostly been described for individual sites or regions, and only rarely investigated on genome-wide scale [23, 25, 31, 44]. Since the exact function of DNA methylation in PCa is still largely unknown, the investigation of the less known patterns and disruptions during carcinogenesis could reveal novel aspects of PCa development and progression. Although downregulation of gene expression was found to be the most pronounced effect of hypermethylation in the present study, we also show that hypermethylation in the promoter region can be associated with upregulation of gene expression. We first observed UPUP pattern in the *Absher* dataset with a limited number of methylation probes, and later demonstrated corresponding results when expanding to HM450 probes in all three datasets — hypermethylation of a large number of probes correlated positively with the expression of their associated genes. We showed that just a few genes in each pattern were associated with both hypomethylated and hypermethylated probes, indicating the robustness of the identified groups of genes. For our top ten UPUP-only and UPDOWN-only genes, *EFS* and *SPARCL1* from the UPDOWN-only group have previously been shown to be suppressed in PCa [45, 46]. In another study, promoter hypermethylation and its negative correlation with the gene expression was demonstrated for genes *TMEM106A* and *SCGB3A1* [47]. The gene *ZAR1* from UPUP-only group has been reported to be hypermethylated and upregulated in neuroblastoma, while *CPT1B* was upregulated in PCa and breast cancer, in which the gene promotes chemo-resistance [48, 49]. Another interesting gene from the UPUP-only group is *GSC* (*Goosecoid*), for which the upregulated expression has been associated with phenotype-specific DNA methylation downstream of the TSS in a PCa mouse model [50]. Same hypermethylation for this gene was also shown in our study (Additional file 1: Figure S2).

We observed a clearly distinguishable and robust UPUP pattern between DNA methylation and gene expression around TSS for associated genes (Figs. 2, 3, 4 and 5), although this pattern was somewhat weaker than the corresponding UPDOWN pattern. Genes in the UPUP group also displayed different functional association compared to UPDOWN genes, demonstrating a potential biological relevance of UPUP regulated genes. Overall, these results strengthen the hypothesis of more complex associations between DNA methylation and gene expression, which should warrant further investigation of genes displaying methylation patterns deviating from the classical UPDOWN pattern.

CGI is a noteworthy feature to be considered in the context of gene regulation, and when CGIs are found in the promoter region near TSSs, such regions are often targets for DNA methylation [23, 26]. In addition, the distance between methylated position and TSS is also an important measure for transcription regulation. Irizarry et al. investigated methylated sites in colon cancer, located 300 bp upstream from TSSs, and demonstrated that methylation of promoters can correlate positively with the expression of a target gene [44]. In our study, more than 85% of all upregulated and downregulated genes have at least one probe within 300 bp from TSS and around a quarter of all probes in each group are located immediately upstream of TSS (Fig. 5, Additional file 1: Table S4, Additional file 1: Figure S1). In addition, even though not with a big difference, UPDOWN-only genes have slightly more hypermethylated probes in first introns and first exons (Table 2). Furthermore, probes are similarly present in CGIs for UPUP-only and UPDOWN-only genes with most of the genes having at least one probe in a CGI or its shore or shelf (Additional file 1: Table S5). Eight and seven out of the top ten upregulated and downregulated genes contained CGIs, respectively. Wan et al. also demonstrated that the genomic locations of the regions were similar for both positively and negatively correlating regions in colon cancer [25]. The literature also states that silencing protein complexes tend to be recruited more often to the CGI-promoters compared with promoters without CGIs [38]. However, it is important to note that there is very little information known about possible gene activation by DNA hypermethylation and what kind of proteins are recruited in these mechanisms. Yet some TFs have been shown to have preferred binding to methylated DNA [28–31]. In addition to the structural similarities and differences between UPUP-only and UPDOWN-only genes, a captivating functional difference was observed in the GSEA. The fact that UPUP-only genes were associated with features of the nuclei — chromatin, DNA packaging and nucleosomes — possibly links these genes to the regulatory programs at the DNA level. UPDOWN-only genes seem more likely to be involved in cell differentiation and phenotype changes, since GSEA showed associations with extracellular features for these genes.

Our study demonstrates that a significant portion of DNA methylation events in PCa can be positively correlated with changes in gene expression. This underlines the importance of epigenetic measurements being accompanied by measurements of gene expression changes to delineate the actual effect of DNA methylation on gene expression. However, some limitations are present in the study. First, prostate cancer tissue samples are heterogeneous, and generally contain a mixture of tissue types. In particular, gene expression changes in PCa studies are known to be influenced by stroma tissue confounding [4, 35]. This is a general problem in this type of tissue sample cohorts, and it is possible that some of the differential expression and methylation changes observed are due to systematic biases in cell-type composition rather than PCa features. However, the presented gene level associations between methylation and gene expression, which we focus on in this study, should be valid regardless of the source of variation. Second, the study relies on methylation of single sites from the 27 K and HM450 methylation arrays. It is likely that expansion of these single sites into differentially methylated regions would shed further light on how individual differentially methylated sites relates to the methylation status of the whole region, and its subsequent effect on gene expression. DNA methylation sequencing or even higher resolution DNA methylation arrays could be used in future studies to increase this precision. At present, genome-wide methylation data on PCa is limited, and rarely comes with corresponding measurements of gene expression. Nevertheless, this study using currently available single-site genome-wide data allows us to robustly detect and shed light on less appreciated relations between DNA methylation and gene expression. This is important in order to bring improvements to the field of epigenetics and cancer research.

## Conclusions

In this study, we show that a significant fraction of hypermethylated genes is associated with upregulated gene expression in prostate cancer. Our analysis of genome-wide data from large cohorts demonstrates that this result is robust and comparable in strength to the canonical association, where hypermethylated genes are associated with gene suppression. Our results therefore explore an additional complexity of epigenetic changes supplementing the classical understanding of regulation by DNA methylation. The findings presented here emphasize the importance of integrating gene expression data in DNA methylation studies to avoid drawing false conclusions about the effect of DNA methylation on gene expression. In this study, we focus on prostate cancer, but the observed relations between DNA hypermethylation and gene expression are likely to be recurrent for other types of cancers and biological systems, which should be a focus for further studies.

## Methods

### Data

#### DNA methylation

DNA methylation datasets for PCa were identified in the Gene Expression Omnibus (GEO) database, using keyword phrase “*prostate cancer methylation*” [51, 52]. We identified 16 datasets containing DNA methylation data from PCa, and two of them (GSE26126 and GSE76938) were selected for the analysis. The third methylation dataset used in the analysis was from the Cancer Genome Atlas (TCGA). Information about all datasets is displayed in Table 1. The criteria for selection was that the cohorts were of sufficient size (> 100 samples), contained both cancer and normal samples, and used comparable platforms for analysis (Illumina BeadChip arrays). The similarity in platform facilitated more easy comparison of methylation sites between the datasets.

#### Gene expression

A previously generated meta-analysis of gene expression changes between cancer and normal samples from five PCa cohorts [35] was used to define genes consistently up- or downregulated in PCa vs normal samples. In addition, TCGA gene expression data was processed and matched to TCGA DNA methylation data to facilitate direct comparison between samples. Samples were matched using TCGA barcodes, and 497 prostate cancer samples and 35 normal tissue samples in TCGA cohort have both DNA methylation and gene expression data.

### Data processing and establishment of groups of gene regulation patterns

The complete workflow of the analysis is shown in Fig. 1. In the first part of the study, three DNA methylation datasets — *Absher*, *Kirby* and *TCGA* — were used. All three methylation datasets were produced using Illumina technology (HM450 or Methylation 27 k), which allowed us to identify corresponding probes in all datasets. Corresponding probes in all three datasets were prioritized for further analysis. Names of genes were matched to probes using the GPL8490–65 reference. Differentially methylated probes were identified using *limma* package in R [53]. The meta expression dataset was used to associate DNA methylation of the genes with gene expression status. Differentially expressed genes were associated with differentially methylated probes (with the same methylation status in all three datasets), based on up/downregulation of methylation and gene expression differences. This resulted in four groups of regulation patterns: UPUP (methylation gain with upregulated gene expression), UPDOWN (methylation gain, downregulated expression), DOWNUP (methylation loss, upregulated expression) and DOWNDOWN (methylation loss, downregulated expression). DNA methylation fold changes for individual genes were calculated by averaging fold changes of corresponding probes in all three DNA methylation datasets. Top 150 genes with highest DNA methylation fold change in each regulation pattern group were selected for gene expression and DNA methylation fold change visualization in a scatterplot. The average DNA methylation fold change for a gene was calculated from all corresponding methylation probes for that gene in *Absher*, *Kirby* and *TCGA* DNA methylation datasets. Further analysis focused on the non-canonical UPUP group and investigating its robustness by comparing it with the classical UPDOWN group.

### Extending the list of methylation probes and re-establishment of the groups of gene regulation patterns

The identification of the initial sets of UPUP and UPDOWN probes and genes were limited to the 27 k promoter regions identified in the *Absher* DNA methylation dataset. In the second part we expanded the analysis using the *TCGA* DNA methylation dataset, increasing the number of probes to 450 k, and used this data to extend the list of probes associated with genes in the UPUP and UPDOWN groups. The GPL13534–11288 reference available at GEO was used to match genes to probes in the *TCGA* dataset. The list of probes for UPUP and UPDOWN genes was then extracted and genes re-grouped according to their methylation status. Genes that are associated with hypermethylated or non-differentially methylated probes, but no hypomethylated probes, were further selected and appointed to two new groups — UPUP-only and UPDOWN-only.

### Correlation of probe methylation with gene expression

In the third part of the study *TCGA combined dataset* was used to correlate methylation profiles of each probe to the expression profiles of its corresponding genes. The Pearson correlation was calculated for all probe to gene association according to the GPL13534–11288 reference. Hypermethylated probes from UPUP-only and UPDOWN-only regulation patterns were grouped according to the correlation score and then calculated. Genes associated with these probes were also counted. We defined 11 correlation groups: very strong negative correlation (score from − 1 to − 0.5), strong negative (− 0.5 to − 0.4), intermediate negative (− 0.4 to − 0.2), weak negative (− 0.2 to − 0.1), very weak negative (− 0.1 to 0), no correlation (score equals to zero), very weak positive correlation (0 to 0.1), weak positive (0.1 to 0.2), intermediate positive (0.2 to 0.4), strong positive (0.4 to 0.5) and very strong positive correlation group (0.5 to 1).

### Functional and structural analysis of the genes and probes in UPUP-only and UPDOWN-only groups and visualization of the most significantly hypermethylated probes

The fourth part of the study focused on specific genomic features, such as transcriptional start sites (TSSs), CpG islands (CGIs), their shores and shelves in order to compare genes from UPUP-only and UPDOWN-only groups. The distances from each probe associated with the genes in each of two analysed patterns were determined using the GPL13534–11288 probe reference. First, the numbers of different TSSs for each gene in the pattern was determined. Secondly, the probes that are in windows of 50, 100, 200, 300, 400, 500, 1000, 1500 and 2000 bp surrounding the TSS were counted. These probes were associated with the genes, and the number of genes for each distance was also determined likewise. Following this, numbers of probes that are located in regions that are in different distances from the TSS were calculated. The regions are − 1500 to − 1000, − 1000 to − 500, − 500 to − 400, − 400 to − 300, − 300 to − 200, − 200 to − 100, − 100 to − 50, − 50 to 0 bp from TSS and same regions upstream TSS, including 1500 to 2000 and 2000 and more bp from TSS. In addition, probes in each region were divided into two groups, based on their methylation fold change. High fold changes raging from 0.5 to 0.2 and low fold changes below 0.2. The genomic location (CGI, its shelf or shore) of each hypermethylated probe was determined using the GPL13534–11288 probe reference. Probes associated with one of these features: N shelf, N shore, CpG island, S shore, S shelf or no feature, were counted and grouped according to the feature. Numbers of genes associated with probes in each group was then determined.

The locations of hypermethylated and non-differentially methylated probes in different gene regions were determined. Gene regions representing 3’UTR, 5’UTR, coding exons, exons and introns from the GRCh38/hg38 human genome reference were downloaded from UCSC Genome Browser with NCBI RefSeq annotation (http://genome.ucsc.edu) [54]. Coordinates of the methylation probes were overlapped with each type of gene region associated with UPUP-only and UPDOWN-only genes. Numbers of probes in each group and for each region were counted and fractions of all significantly hypermethylated or non-differentially hypermethylated probes were calculated. In addition, the fraction of overlapping exon/coding exon/intron probes located in the first exon/coding exon/intron was calculated.

Furthermore, gene set enrichment analysis was used to investigate possible functional properties of the two lists of genes. Genes in the UPUP-only and UPDOWN-only regulatory groups were used as an input in Enrichr web-based tool [55, 56].

Finally, the focus from genome-wide analysis was shifted to single cases by visualizing the probes associated with UPUP-only and UPDOWN-only genes. The average *p*-values for all significantly hypermethylated probes associated with genes following UPUP-only and UPDOWN-only patterns were calculated in order to distinguish top 10 genes associated with the most significantly hypermethylated probes in each pattern. Positions of both significantly hypermethylated and non-differentially methylated probes were determined using GRCh38/hg38 human genome assembly and displayed in UCSC Genome Browser (http://genome.ucsc.edu) [54]. Additionally, top 10 genes were searched in a DataBase of Transcriptional Start Sites (DBTSS, http://dbtss.hgc.jp) [57, 58] and in ZENBU genome browser, based on FANTOM data (http://fantom.gsc.riken.jp/zenbu/) [59, 60]. DBTSS and ZENBU gives an overview of different TSSs in various human tissues and cell lines.

## Supplementary information

**Additional file 1: Table S1.** Numbers of genes in three datasets Absher, Kirby and TCGA (with overlap) that can be assigned to the four groups of regulation patterns, based on DNA methylation of the probes and expression of the associated gene: UPUP (gain of methylation — upregulated expression), UPDOWN (gain of methylation — downregulated expression), DOWNUP (loss of methylation — upregulated expression) and DOWNDOWN (loss of methylation — downregulated expression). **Table S2.** Number of genes, associated with multiple probes, in Absher, Kirby and TCGA datasets for all four established groups of gene regulation patterns: UPUP, UPDOWN, DOWNUP and DOWNDOWN. Genes that are associated with both hypermethylated and hypomethylated probes are defined as inconsistent and indicated in the table. **Table S3.** Probes in TCGA combined methylation/gene expression dataset for UPUP-only and UPDOWN-only groups that can be assigned to different groups, according to their probe DNA methylation and associated gene expression correlation. In addition, number of genes, associated with the probes in each correlation group, are displayed. **Table S4.** Number of probes in the TCGA methylation dataset for UPUP-only and UPDOWN-only groups that are located in different distances from the TSSs of the associated genes. **Table S5.** Number of probes in the TCGA DNA methylation dataset for UPUP-only and UPDOWN-only regulation pattern groups that are located in CGIs, their shores, shelves or all locations. Number of genes that the probes can be associated with is also displayed. **Table S6.** Top 10 most significantly hypermethylated genes in UPUP-only and UPDOWN-only regulation pattern groups. **Figure S1.** Hypermethylated probes associated with genes following UPUP-only and UPDOWN-only are located 50 to 2000 bp upstream or downstream from the TSS of the genes. **Figure S2.** UCSC Genome Browser window for the gene GSC (Goosecoid) together with methylation fold changes for each visualized position in the same order.

## Data Availability

The methylation datasets and TCGA gene expression dataset analysed during the current study are available in the Gene Expression Omnibus (GEO) and the Cancer Genome Atlas (TCGA) repositories: https://www.ncbi.nlm.nih.gov/geo/query/acc.cgi?acc=GSE26126, https://www.ncbi.nlm.nih.gov/geo/query/acc.cgi, https://portal.gdc.cancer.gov. The meta-analysis of gene expression was from the publication referenced in [35].

## References

[1] Ferlay J, Soerjomataram I, Dikshit R, Eser S, Mathers C, Rebelo M, Parkin DM, Forman D, Bray F (2015). Cancer incidence and mortality worldwide: sources, methods and major patterns in GLOBOCAN 2012. Int J Cancer.

[2] Cancer in Norway 2016 — Cancer incidence, mortality, survival and prevalence in Norway [https://www.kreftregisteret.no/en/].

[3] Siegel RL, Miller KD, Jemal A (2018). Cancer statistics, 2018. CA Cancer J Clin.

[4] Kobayashi M, Ishida H, Shindo T, Niwa S, Kino M, Kawamura K, Kamiya N, Imamoto T, Suzuki H, Hirokawa Y (2008). Molecular analysis of multifocal prostate cancer by comparative genomic hybridization. Prostate.

[5] Armenia J, Wankowicz SAM, Liu D, Gao J, Kundra R, Reznik E, Chatila WK, Chakravarty D, Han GC, Coleman I (2018). The long tail of oncogenic drivers in prostate cancer. Nat Genet.

[6] Robinson D, Van Allen EM, Wu YM, Schultz N, Lonigro RJ, Mosquera JM, Montgomery B, Taplin ME, Pritchard CC, Attard G (2015). Integrative clinical genomics of advanced prostate cancer. Cell.

[7] Massie CE, Mills IG, Lynch AG (2017). The importance of DNA methylation in prostate cancer development. J Steroid Biochem Mol Biol.

[8] Long Mark, Smiraglia Dominic, Campbell Moray (2017). The Genomic Impact of DNA CpG Methylation on Gene Expression; Relationships in Prostate Cancer. Biomolecules.

[9] Witte T, Plass C, Gerhauser C (2014). Pan-cancer patterns of DNA methylation. Genome Med.

[10] Bibikova M, Barnes B, Tsan C, Ho V, Klotzle B, Le JM, Delano D, Zhang L, Schroth GP, Gunderson KL (2011). High density DNA methylation array with single CpG site resolution. Genomics.

[11] Gardiner-Garden M, Frommer M (1987). CpG islands in vertebrate genomes. J Mol Biol.

[12] Friedlander TW, Roy R, Tomlins SA, Ngo VT, Kobayashi Y, Azameera A, Rubin MA, Pienta KJ, Chinnaiyan A, Ittmann MM (2012). Common structural and epigenetic changes in the genome of castration-resistant prostate cancer. Cancer Res.

[13] Lim DHK, Maher ER (2010). DNA methylation: a form of epigenetic control of gene expression. Obstet Gynaecol.

[14] Esteller M (2002). CpG island hypermethylation and tumor suppressor genes: a booming present, a brighter future. Oncogene.

[15] Ehrlich M (2009). DNA hypomethylation in cancer cells. Epigenomics.

[16] Ehrlich M (2002). DNA methylation in cancer: too much, but also too little. Oncogene.

[17] Morey SR, Smiraglia DJ, James SR, Yu J, Moser MT, Foster BA, Karpf AR (2006). DNA methylation pathway alterations in an autochthonous murine model of prostate cancer. Cancer Res.

[18] Laird PW (2003). The power and the promise of DNA methylation markers. Nat Rev Cancer.

[19] Kirby MK, Ramaker RC, Roberts BS, Lasseigne BN, Gunther DS, Burwell TC, Davis NS, Gulzar ZG, Absher DM, Cooper SJ (2017). Genome-wide DNA methylation measurements in prostate tissues uncovers novel prostate cancer diagnostic biomarkers and transcription factor binding patterns. BMC Cancer.

[20] Vandekerkhove G, Chi KN, Wyatt AW (2018). Clinical utility of emerging liquid biomarkers in advanced prostate cancer. Cancer Gene Ther.

[21] Yang M, Park JY (2012). DNA methylation in promoter region as biomarkers in prostate cancer. Methods Mol Biol.

[22] Kobayashi Y, Absher DM, Gulzar ZG, Young SR, McKenney JK, Peehl DM, Brooks JD, Myers RM, Sherlock G (2011). DNA methylation profiling reveals novel biomarkers and important roles for DNA methyltransferases in prostate cancer. Genome Res.

[23] Marzese DM, Hoon DS (2015). Emerging technologies for studying DNA methylation for the molecular diagnosis of cancer. Expert Rev Mol Diagn.

[24] Medvedeva Yulia A, Khamis Abdullah M, Kulakovskiy Ivan V, Ba-Alawi Wail, Bhuyan Md Shariful I, Kawaji Hideya, Lassmann Timo, Harbers Matthias, Forrest Alistair RR, Bajic Vladimir B (2014). Effects of cytosine methylation on transcription factor binding sites. BMC Genomics.

[25] Wan J, Oliver VF, Wang G, Zhu H, Zack DJ, Merbs SL, Qian J (2015). Characterization of tissue-specific differential DNA methylation suggests distinct modes of positive and negative gene expression regulation. BMC Genomics.

[26] Siegfried Z, Simon I (2010). DNA methylation and gene expression. Wiley Interdiscip Rev Syst Biol Med.

[27] Furst RW, Kliem H, Meyer HH, Ulbrich SE (2012). A differentially methylated single CpG-site is correlated with estrogen receptor alpha transcription. J Steroid Biochem Mol Biol.

[28] Zhu H, Wang G, Qian J (2016). Transcription factors as readers and effectors of DNA methylation. Nat Rev Genet.

[29] Yin Yimeng, Morgunova Ekaterina, Jolma Arttu, Kaasinen Eevi, Sahu Biswajyoti, Khund-Sayeed Syed, Das Pratyush K., Kivioja Teemu, Dave Kashyap, Zhong Fan, Nitta Kazuhiro R., Taipale Minna, Popov Alexander, Ginno Paul A., Domcke Silvia, Yan Jian, Schübeler Dirk, Vinson Charles, Taipale Jussi (2017). Impact of cytosine methylation on DNA binding specificities of human transcription factors. Science.

[30] Hu S, Wan J, Su Y, Song Q, Zeng Y, Nguyen HN, Shin J, Cox E, Rho HS, Woodard C (2013). DNA methylation presents distinct binding sites for human transcription factors. Elife.

[31] Rishi V, Bhattacharya P, Chatterjee R, Rozenberg J, Zhao J, Glass K, Fitzgerald P, Vinson C (2010). CpG methylation of half-CRE sequences creates C/EBPalpha binding sites that activate some tissue-specific genes. Proc Natl Acad Sci U S A.

[32] Weber M, Hellmann I, Stadler MB, Ramos L, Paabo S, Rebhan M, Schubeler D (2007). Distribution, silencing potential and evolutionary impact of promoter DNA methylation in the human genome. Nat Genet.

[33] Boyes J, Bird A (1992). Repression of genes by DNA methylation depends on CpG density and promoter strength: evidence for involvement of a methyl-CpG binding protein. EMBO J.

[34] Cancer Genome Atlas Research N (2015). The molecular taxonomy of primary prostate Cancer. Cell.

[35] Rye MB, Bertilsson H, Andersen MK, Rise K, Bathen TF, Drablos F, Tessem MB (2018). Cholesterol synthesis pathway genes in prostate cancer are transcriptionally downregulated when tissue confounding is minimized. BMC Cancer.

[36] Park JY (2010). Promoter hypermethylation in prostate cancer. Cancer Control.

[37] Kim JH, Dhanasekaran SM, Prensner JR, Cao X, Robinson D, Kalyana-Sundaram S, Huang C, Shankar S, Jing X, Iyer M (2011). Deep sequencing reveals distinct patterns of DNA methylation in prostate cancer. Genome Res.

[38] O'Hagan HM, Wang W, Sen S, Destefano Shields C, Lee SS, Zhang YW, Clements EG, Cai Y, Van Neste L, Easwaran H (2011). Oxidative damage targets complexes containing DNA methyltransferases, SIRT1, and polycomb members to promoter CpG Islands. Cancer Cell.

[39] Cao R, Zhang Y (2004). SUZ12 is required for both the histone methyltransferase activity and the silencing function of the EED-EZH2 complex. Mol Cell.

[40] Ren G, Baritaki S, Marathe H, Feng J, Park S, Beach S, Bazeley PS, Beshir AB, Fenteany G, Mehra R (2012). Polycomb protein EZH2 regulates tumor invasion via the transcriptional repression of the metastasis suppressor RKIP in breast and prostate cancer. Cancer Res.

[41] Jain P, Di Croce L (2016). Mutations and deletions of PRC2 in prostate cancer. Bioessays.

[42] Chase A, Cross NC (2011). Aberrations of EZH2 in cancer. Clin Cancer Res.

[43] Vire E, Brenner C, Deplus R, Blanchon L, Fraga M, Didelot C, Morey L, Van Eynde A, Bernard D, Vanderwinden JM (2006). The Polycomb group protein EZH2 directly controls DNA methylation. Nature.

[44] Irizarry RA, Ladd-Acosta C, Wen B, Wu Z, Montano C, Onyango P, Cui H, Gabo K, Rongione M, Webster M (2009). The human colon cancer methylome shows similar hypo- and hypermethylation at conserved tissue-specific CpG island shores. Nat Genet.

[45] Sertkaya S, Hamid SM, Dilsiz N, Varisli L (2015). Decreased expression of EFS is correlated with the advanced prostate cancer. Tumour Biol.

[46] Xiang Y, Qiu Q, Jiang M, Jin R, Lehmann BD, Strand DW, Jovanovic B, DeGraff DJ, Zheng Y, Yousif DA (2013). SPARCL1 suppresses metastasis in prostate cancer. Mol Oncol.

[47] Geybels MS, Zhao S, Wong CJ, Bibikova M, Klotzle B, Wu M, Ostrander EA, Fan JB, Feng Z, Stanford JL (2015). Epigenomic profiling of DNA methylation in paired prostate cancer versus adjacent benign tissue. Prostate.

[48] Sugito K, Kawashima H, Yoshizawa S, Uekusa S, Hoshi R, Furuya T, Kaneda H, Hosoda T, Konuma N, Masuko T (2013). Non-promoter DNA hypermethylation of zygote arrest 1 (ZAR1) in neuroblastomas. J Pediatr Surg.

[49] Wang T, Fahrmann JF, Lee H, Li YJ, Tripathi SC, Yue C, Zhang C, Lifshitz V, Song J, Yuan Y (2018). JAK/STAT3-regulated fatty acid beta-oxidation is critical for breast Cancer stem cell self-renewal and Chemoresistance. Cell Metab.

[50] Camoriano M, Kinney SR, Moser MT, Foster BA, Mohler JL, Trump DL, Karpf AR, Smiraglia DJ (2008). Phenotype-specific CpG island methylation events in a murine model of prostate cancer. Cancer Res.

[51] Barrett T, Wilhite SE, Ledoux P, Evangelista C, Kim IF, Tomashevsky M, Marshall KA, Phillippy KH, Sherman PM, Holko M (2013). NCBI GEO: archive for functional genomics data sets--update. Nucleic Acids Res.

[52] Edgar R, Domrachev M, Lash AE (2002). Gene expression omnibus: NCBI gene expression and hybridization array data repository. Nucleic Acids Res.

[53] Smyth G, Gentleman R, Carey V, Dudoit S, Irizarry R, Huber W (2005). Limma: linear models for microarray data. Bioinformatics and Computational Biology Solutions using R and Bioconductor.

[54] Kent WJ, Sugnet CW, Furey TS, Roskin KM, Pringle TH, Zahler AM, Haussler D (2002). The human genome browser at UCSC. Genome Res.

[55] Chen EY, Tan CM, Kou Y, Duan Q, Wang Z, Meirelles GV, Clark NR, Ma'ayan A (2013). Enrichr: interactive and collaborative HTML5 gene list enrichment analysis tool. BMC Bioinformatics.

[56] Kuleshov MV, Jones MR, Rouillard AD, Fernandez NF, Duan Q, Wang Z, Koplev S, Jenkins SL, Jagodnik KM, Lachmann A (2016). Enrichr: a comprehensive gene set enrichment analysis web server 2016 update. Nucleic Acids Res.

[57] Suzuki A, Wakaguri H, Yamashita R, Kawano S, Tsuchihara K, Sugano S, Suzuki Y, Nakai K (2015). DBTSS as an integrative platform for transcriptome, epigenome and genome sequence variation data. Nucleic Acids Res.

[58] Yamashita R, Sugano S, Suzuki Y, Nakai K (2012). DBTSS: DataBase of transcriptional start sites progress report in 2012. Nucleic Acids Res.

[59] Consortium F, Forrest AR, Kawaji H, Rehli M, Baillie JK, de Hoon MJ, Haberle V, Lassmann T, the RP, Clst (2014). A promoter-level mammalian expression atlas. Nature.

[60] Severin J, Lizio M, Harshbarger J, Kawaji H, Daub CO, Hayashizaki Y, Consortium F, Bertin N, Forrest AR (2014). Interactive visualization and analysis of large-scale sequencing datasets using ZENBU. Nat Biotechnol.

