# Supplementary material for: DNA hypermethylation associated with upregulated gene expression in prostate cancer demonstrates the diversity of epigenetic regulation
Source: BMC Med Genomics. 2020 Jan 8;13:6. doi: 10.1186/s12920-020-0657-6 (PMC6950795; doi:10.1186/s12920-020-0657-6)
Supplement: Supplementary file 1 — Additional file 1: Table S1. Numbers of genes in three datasets Absher, Kirby and TCGA (with overlap) that can be assigned to the four groups of regulation patterns, based on DNA methylation of the probes and expression of the associated gene: UPUP (gain of methylation — upregulated expression), UPDOWN (gain of methylation — downregulated expression), DOWNUP (loss of methylation — upregulated expression) and DOWNDOWN (loss of methylation — downregulated expression). Table S2. Number of genes, associated with multiple probes, in Absher, Kirby and TCGA datasets for all four established groups of gene regulation patterns: UPUP, UPDOWN, DOWNUP and DOWNDOWN. Genes that are associated with both hypermethylated and hypomethylated probes are defined as inconsistent and indicated in the table. Table S3. Probes in TCGA combined methylation/gene expression dataset for UPUP-only and UPDOWN-only groups that can be assigned to different groups, according to their probe DNA methylation and associated gene expression correlation. In addition, number of genes, associated with the probes in each correlation group, are displayed. Table S4. Number of probes in the TCGA methylation dataset for UPUP-only and UPDOWN-only groups that are located in different distances from the TSSs of the associated genes. Table S5. Number of probes in the TCGA DNA methylation dataset for UPUP-only and UPDOWN-only regulation pattern groups that are located in CGIs, their shores, shelves or all locations. Number of genes that the probes can be associated with is also displayed. Table S6. Top 10 most significantly hypermethylated genes in UPUP-only and UPDOWN-only regulation pattern groups. Figure S1. Hypermethylated probes associated with genes following UPUP-only and UPDOWN-only are located 50 to 2000 bp upstream or downstream from the TSS of the genes. Figure S2. UCSC Genome Browser window for the gene GSC (Goosecoid) together with methylation fold changes for each visualized position in the same order. [file 12920_2020_657_MOESM1_ESM.pdf]

DNA HYPERMETHYLATION ASSOCIATED WITH UPREGULATED GENE EXPRESSION IN PROSTATE CANCER DEMONSTRATES THE DIVERSITY OF EPIGENETIC REGULATION

Ieva Rauluseviciute[1*], Finn Drabløs[1], Morten Beck Rye[1,2]

[1] Department of Clinical and Molecular Medicine, NTNU - Norwegian University of Science and Technology, NO-7491 Trondheim, Norway

[2] Clinic of Surgery, St. Olavs Hospital, Trondheim University Hospital, NO-7030 Trondheim, Norway

[*] Corresponding author:

Ieva Rauluseviciute
Department of Cancer Research and Molecular Medicine
Norwegian University of Science and Technology
P.O. Box 8905
NO-7491 Trondheim
Norway
Email: ieva.rauluseviciute@gmail.com
Phone: +4796707578

ADDITIONAL FILE 1

Tables S1 – S6 and Figures S1 and S2

Table S1 **Numbers of genes in three datasets** ***Absher*****,** ***Kirby*** **and** ***TCGA*** **(with overlap) that can be assigned to the four groups of regulation patterns, based on DNA methylation of the probes and expression of the associated gene: UPUP (gain of methylation — upregulated expression), UPDOWN (gain of methylation — downregulated expression), DOWNUP (loss of methylation — upregulated expression) and DOWNDOWN (loss of methylation — downregulated expression).** The numbers in brackets specify what part of all genes for each pattern and in a particular dataset is an overlap.

	Absher	***Kirby***	***TCGA***	**OVERLAP**
**UPUP**	880 (81.02%)	965 (73.89%)	843 (84.58%)	713
**UPDOWN**	1641 (89.95%)	1721 (85.76%)	1618 (91.22%0	1476
**DOWNUP**	557 (88.33%)	630 (78.10%)	891 (55.22%)	492
**DOWNDOWN**	394 (84.77%)	444 (75.23%)	630 (53.02%)	334

Table S2 **Number of genes, associated with multiple probes, in *Absher*, *Kirby* and *TCGA* datasets for all four established groups of gene regulation patterns: UPUP, UPDOWN, DOWNUP and DOWNDOWN. Genes that are associated with both hypermethylated and hypomethylated probes are defined as inconsistent and indicated in the table.** The numbers in brackets specify what part of all genes, associated with multiple probes, which are inconsistent genes.

		Absher	***Kirby***	***TCGA***
**UPUP**	**Genes associated with multiple probes**	216	325	258
	**Inconsistent genes**	2 genes (0.93% of all genes with multiple probes) *GNAS*, *MGMT*	3 genes (0.92%) *ATP10A*, *GNAS*, *NTM*	5 genes (1.94%) *CHFR*, *GNAS*, *MGMT*, *NTM*, *PRR4*
**UPDOWN**	**Genes associated with multiple probes**	589	782	720
	**Inconsistent genes**	3 genes (0.51%) *BCL2*, *CCND1*, *PEG10*	8 genes (1.02%) *CCND1*, *IGF2*, *INS-IGF2*, *PEG10*, *RAB32*, *SEMA3B*, *SGCE*, *ZIM2*	9 genes (1.25%) *BCL2*, *C21orf29*, *GNASAS*, *IGF2*, *INS-IGF2*, *MEG3*, *NTRK1*, *PEG10*, *SGCE*
**DOWNUP**	**Genes associated with multiple probes**	89	149	211
	**Inconsistent genes**	2 genes (2.25%) *GNAS*, *MGMT*	3 genes (2.01%) *ATP10A*, *GNAS*, *NTM*	5 genes (2.37%) *CHFR*, *GNAS*, *MGMT*, *NTM*, *PRR4*
**DOWNDOWN**	**Genes associated with multiple probes**	57	91	141
	**Inconsistent genes**	3 genes (5.26%) *BCL2*, *CCND1*, *PEG10*	8 genes (8.79%) *CCND1*, *IGF2*, *INS-IGF2*, *PEG10*, *RAB32*, *SEMA3B*, *SGCE*, *ZIM2*	9 genes (6.38%) *BCL2*, *C21orf29*, *GNASAS*, *IGF2*, *INS-IGF2*, *MEG3*, *NTRK1*, *PEG10*, *SGCE*

Table S3 **Probes in *TCGA combined* methylation/gene expression dataset for UPUP-only and UPDOWN-only groups that can be assigned to different groups, according to their probe DNA methylation and associated gene expression correlation. In addition, number of genes, associated with the probes in each correlation group, are displayed.** Correlation groups are named from 'Very strong negative' to 'No correlation' to 'Very strong positive'. Different correlation scores (b) are defined for each correlation group.

**Correlation group**	**Correlation score (b)**	**UPUP-only** *105 genes* *1424 probes*		**UPDOWN-only** *192 genes* *2664 probes*	
		**Probes**	**Genes**	**Probes**	**Genes**
**Very strong negative**	**-1 ≤ b < -0.5**	13 (0.91% of all correlating probes)	7 (6.67% of all genes)	240 (9.01%)	57 (29.69%)
**Strong negative**	**-0.5 ≤ b < -0.4**	36 (2.53%)	12 (11.43%)	190 (7.13%)	79 (41.15%)
**Intermediate negative**	**-0.4 ≤ b < -0.2**	120 (8.43%)	33 (31.43%)	723 (27.14%)	148 (77.08%)
**Weak negative**	**-0.2 ≤ b < -0.1**	183 (12.85%)	55 (52.38%)	562 (21.10%)	143 (74.48%)
**Very weak negative**	**-0.1 ≤ b < 0**	391 (27.46%)	80 (76.19%)	634 (23.80%)	133 (69.27%)
**No correlation**	**b = 0**	0	0	0	0
**Very weak positive**	**0 < b ≤ 0.1**	249 (17.49%)	73 (69.52%)	257 (9.65%)	98 (51.04%)
**Weak positive**	**0.1 < b ≤ 0.2**	184 (12.92%)	48 (45.71%)	46 (1.73%)	29 (15.10%)
**Intermediate positive**	**0.2 < b ≤ 0.4**	216 (15.17%)	33 (31.43%)	9 (0.34%)	5 (2.60%)
**Strong positive**	**0.4 < b ≤ 0.5**	19 (1.33%)	8 (7.62%)	0	0
**Very strong positive**	**0.5 < b ≤ 1**	23 (1.62%)	6 (5.71%)	3 (0.11%)	2 (1.04%)

Table S4 **Number of probes in the *TCGA* methylation dataset for UPUP-only and UPDOWN-only groups that are located in different distances from the TSSs of the associated genes.**

**Distance to the TSS, bp**	**UPUP-only**		**UPDOWN-only**	
	**Methylation fold change 0.5 to 0.2**	**Methylation fold change 0.2 to 0.0**	**Methylation fold change 0.5 to 0.2**	**Methylation fold change 0.2 to 0.0**
**-1500 to -1000**	22 probes (2.22% of all UPUP-only probes)	62 (6.26%)	16 probes (0.84% of all UPDOWN-only probes)	74 (3.90%)
**-1000 to -500**	19 (1.92%)	59 (5.95%)	26 (1.37%)	135 (7.11%)
**-500 to -400**	3 (0.30%)	18 (1.82%)	14 (0.74%)	40 (2.11%)
**-400 to -300**	2 (0.20%)	27 (2.72%)	20 (1.05%)	49 (2.58%)
**-300 to -200**	4 (0.40%)	24 (2.42%)	33 (1.74%)	62 (3.27%)
**-200 to -100**	20 (2.02%)	68 (6.86%)	49 (2.58%)	104 (5.48%)
**-100 to -50**	9 (0.91%)	46 (4.64%)	31 (1.63%)	85 (4.48%)
**-50 to 0**	13 (1.31%)	61 (6.16%)	55 (2.90%)	110 (5.80%)
**0 to 50**	17 (1.72%)	36 (3.63%)	52 (2.74%)	96 (5.06%)
**50 to 100**	6 (0.61%)	25 (2.52%)	30 (1.58%)	59 (3.11%)
**100 to 200**	11 (1.11%)	41 (4.14%)	25 (1.32%)	62 (3.27%)
**200 to 300**	8 (0.81%)	23 (2.32%)	19 (1.00%)	61 (3.21%)
**300 to 400**	4 (0.40%)	30 (3.03%)	19 (1.00%)	38 (2.00%)
**400 to 500**	6 (0.61%)	15 (1.51%)	10 (0.53%)	33 (1.74%)
**500 to 1000**	19 (1.92%)	49 (4.94%)	39 (2.05%)	112 (5.90%)
**1000 to 1500**	23 (2.32%)	39 (3.94%)	15 (0.79%)	60 (3.16%)

**1500 to 2000**	12<br>(1.21%)	24<br>(2.42%)	8<br>(0.42%)	58<br>(3.06%)
**2000 and more**	41<br>(4.14%)	105<br>(10.60%)	17<br>(0.90%)	182<br>(9.59%)

Table S5 **Number of probes in the *TCGA* DNA methylation dataset for UPUP-only and UPDOWN-only regulation pattern groups that are located in CGIs, their shores, shelves or all locations. Number of genes that the probes can be associated with is also displayed.**

**Genomic location**	**UPUP-only**		**UPDOWN-only**	
	**Probes**	**Genes**	**Probes**	**Genes**
**CGI**	524 (52.88% of all probes)	72 (68.57% of all genes)	827 (43.60% of all probes)	125 (65.10% of all genes)
**Shore**	308 (31.08%)	73 (69.52%)	590 (31.10%)	128 (66.67%)
**Shelf**	34 (3.43%)	13 (12.38%)	67 (3.53%)	33 (17.19%)
**CGI, shelf and shore**	-	5 (4.76%)	-	25 (13.02%)

Table S6 **Top 10 most significantly hypermethylated genes in UPUP-only and UPDOWN-only regulation pattern groups.** Listed from the most (1) to the least significantly hypermethylated (10).

Nr.	UPUP-only	UPDOWN-only
**1.**	*CPT1B*	*SCGB3A1*
**2.**	*LTK*	*EFS*
**3.**	*GSC*	*KLF8*
**4.**	*TSPAN16*	*COL3A1*
**5.**	*ZAR1*	*TMEM106A*
**6.**	*TLX1*	*RGN*
**7.**	*FEV*	*SPARCL1*
**8.**	*SRPX2*	*WFDC2*
**9.**	*LRRC25*	*PLA2G3*
**10.**	*HIST1H3E*	*MFAP4*

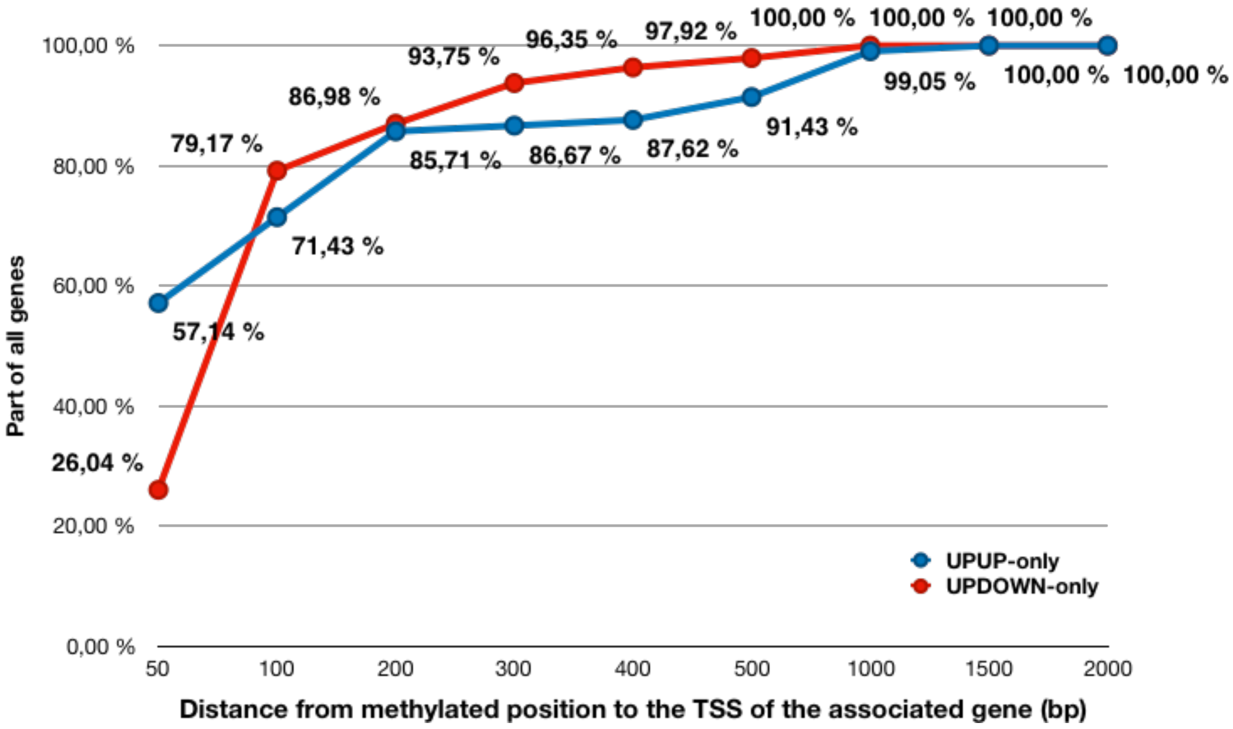

Figure S1 **Hypermethylated probes associated with genes following UPUP-only and UPDOWN-only are located 50 to 2000 bp upstream or downstream from the TSS of the genes.** More genes in UPUP-only regulation pattern have probes within 50 bp from the TSS, whereas more genes in the UPDOWN-only pattern have probes within 100 to 1000 bp from the TSSs.

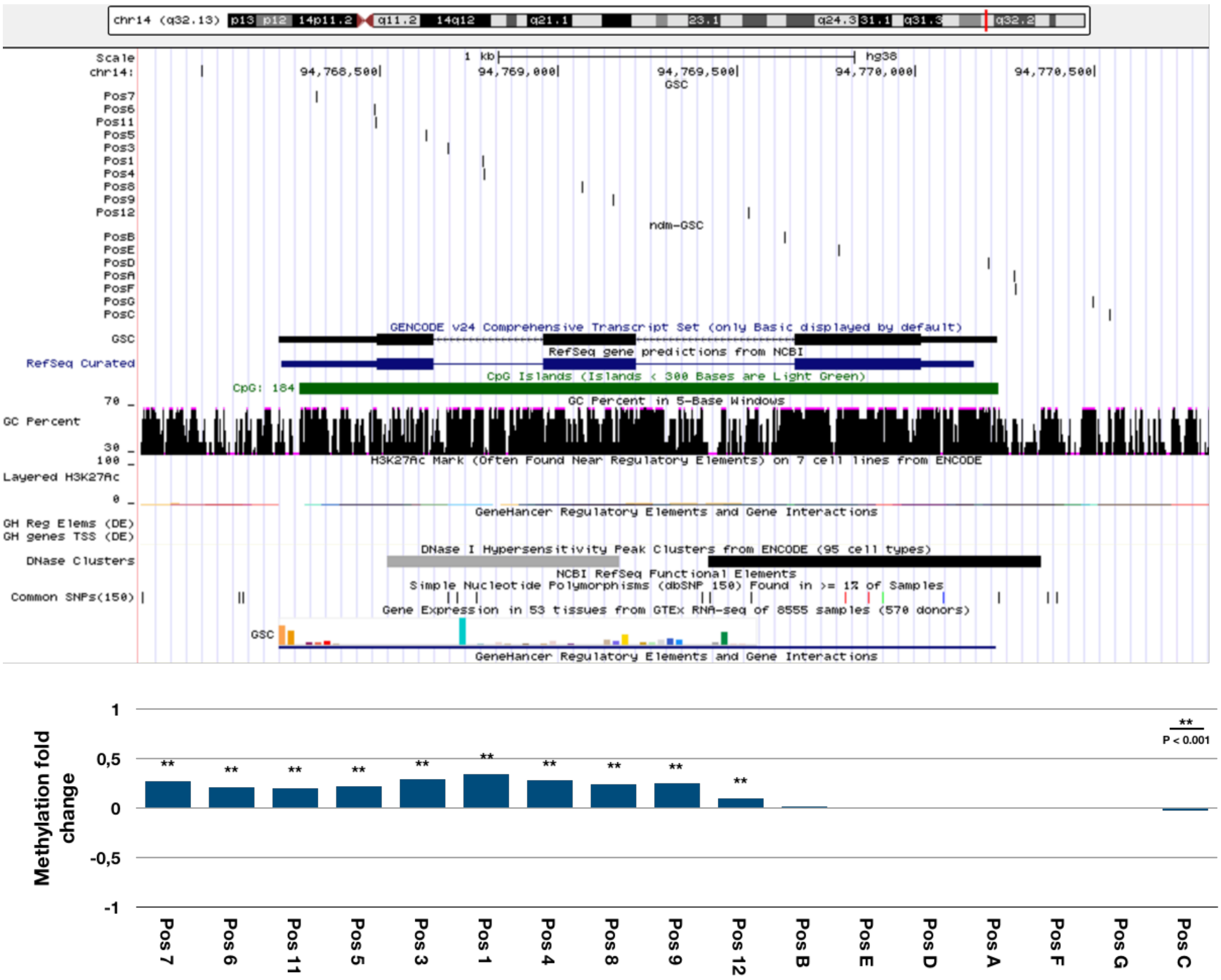

Figure S2 **UCSC Genome Browser window for the gene *GSC* (*Goosecoid*) together with methylation fold changes for each visualized position in the same order.** Significant hypermethylation occurs specifically downstream of the gene. Pos1-12 stand for significantly ($p < 0.001$) hypermethylated probes from most (Pos1) to least (Pos12) significantly methylated. PosA-G are non-differentially methylated probes.
